# Identification of the key genes and pathways involved in B cells in primary Sjögren’ s syndrome

**DOI:** 10.1080/21655979.2021.1930753

**Published:** 2021-05-26

**Authors:** Shizhen Lei, Yi Zhang

**Affiliations:** a Department of Ophthalmology, The Fourth Affiliated Hospital of China Medical University, Shenyang, China; b Department of Gerontology and Geriatrics, Shengjing Hospital of China Medical University, Shenyang, China

**Keywords:** B cell, primary Sjögren’ s syndrome, CIBERSORT, WGCNA, GEO

## Abstract

Primary Sjögren’ s syndrome (pSS) is a relatively common autoimmune disease, which mainly involves the exocrine glands, causing dry eye, dryness of mouth, fatigue and pain in the joints, thus severely affecting the normal lives of patients. B cell populations are considered to play an important role in their pathogenesis and pSS patients are generally characterized by exhibiting biological signs of B cell activation. Moreover, another important characterized change in the peripheral blood of pSS patients is found to be the decreased number of circulating memory B cells. However, the mechanisms underlying the B cell activation and the decreased level of circulating memory B cells in pSS patients are still unclear. Therefore, we identified key genes and pathways involved in B cells in pSS through a combination of several bioinformatic approaches including Cell-type Identification By Estimating Relative Subsets Of RNA Transcripts (CIBERSORT) and weighted gene co-expression network analysis (WGCNA) using gene expression data of pSS patients and controls from an open database Gene Expression Omnibus (GEO). The results may provide some novel insights into the pathogenesis of pSS. Moreover, we constructed and validated a diagnostic model for pSS by using the expression patterns of these key genes, which may assist clinicians in diagnosing pSS.

## Graphical abstract

**Figure:**
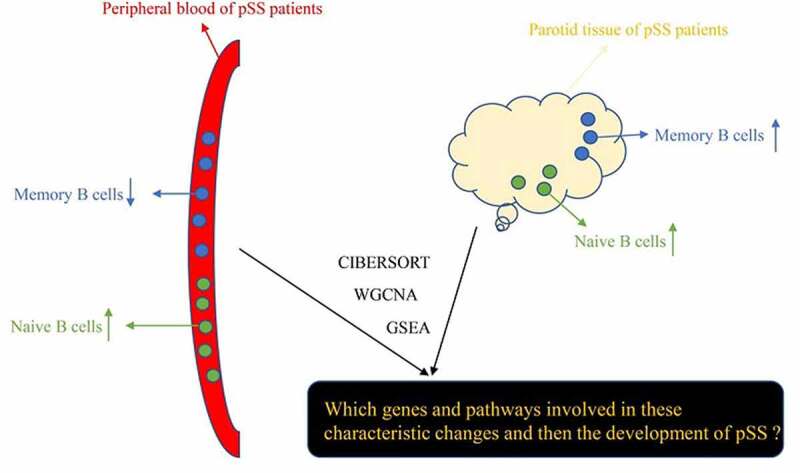

## 1. Introduction

Primary Sjögren’s syndrome (pSS) is a relatively common autoimmune disease preferentially affecting women, the prevalence of which is about 0.3–3 per 1,000 globally [1,2]. pSS is characterized by the infiltration of lymphocytes in exocrine glands including lachrymal and salivary glands, which leads to dryness of eyes and mouth [3]. Moreover, one-third of patients develop systemic complications such as interstitial nephritis and peripheral neuropathy. Notably, B cells have been identified to be significantly involved in the pathogenesis of pSS. pSS patients generally show signs of B cell activation, especially comprising the positivity of autoantibodies, such as anti-Sjögren-syndrome-related antigen A or B antibodies [4]. Moreover, these autoantibodies that specifically occur in pSS have been found to exist long before the symptoms of pSS emerge, which indicates that B cells play an important role in the early development of pSS [5,6]. The levels of B cell subsets in the whole peripheral blood and target organs such as salivary glands of pSS patients have been evaluated and it has been found that the best-characterized change of B cells in pSS patients is the decreased number of memory B cells in the peripheral blood [7–12]. There is one explanation for this phenomenon, which is the migration to or retention of circulating memory B cells within target organs of pSS such as salivary glands [13]. However, to date, the mechanism underlying this change of B cell level is still unknown. As B cell populations are significantly involved in the development of pSS, understanding the mechanisms underlying the activation of B cells and the altered number of B cells in peripheral blood and target organs in pSS patients is urgently needed.

CIBERSORT (Cell-type Identification By Estimating Relative Subsets Of RNA Transcripts) is a robust tool for investigating the proportions of immune cells in obtained samples, such as blood and tissues by using gene expression profile [14]. Although CIBERSORT is often used to assess the immune infiltration in tumor tissues, it also can be used in investigating the non-tumor samples, such as peripheral blood [15,16]. WGCNA (weighted gene co-expression network analysis) is an algorithm frequently used to analyze the expression patterns of multiple genes and the connection between genes and clinical traits [17]. Notably, there are several studies that investigated the mechanism and characteristics of pSS by using RNA sequencing and bioinformatic analysis including WGCNA [18–20], and these studies also inspired our research. In our study, WGCNA was used to identify the co-expression modules significantly related to levels of naïve B cells and memory B cells in whole peripheral blood and parotid tissue from pSS patients. Moreover, we identified the hub genes significantly associated with the levels of B cells by using the String database and Cytoscape software. To the best of our knowledge, the present study is the first to identify the key genes and pathways involved in the activation of B cells and the altered levels of B cells in pSS by the combination of CIBERSORT and WGCNA analysis. Furthermore, we applied Gene Set Enrichment Analysis (GSEA) to find out the Kyoto Encyclopedia of Genes and Genomes (KEGG) pathways enriched in the peripheral blood of pSS patients.

In summary, this study aimed to identify key genes and pathways involved in B cells in pSS through a combination of several bioinformatic approaches, hoping to provide some new insights into the understanding of development and progression of pSS.

## 2. Material and methods

### 2.1. Data collection and preprocessing

We downloaded GSE66795, GSE40611 and GSE51092 datasets from Gene Expression Omnibus (GEO) database (https://www.ncbi.nlm.nih.gov/geo/). GSE66795 dataset contains gene expression profiles of whole peripheral blood samples from 131 pSS patients and 29 healthy controls, and GSE51092 dataset includes gene expression data of whole peripheral blood samples from 190 pSS patients and 32 controls. GSE40611 contains gene expression data of parotid tissues from 17 pSS patients and 18 controls. The gene expression data of these three datasets were firstly standardized through the limma package [21] in R software; then, the ‘ComBat’ algorithm of sva package [22] was used to correct the batch effects in these three datasets caused by non-biotechnological bias.

### 2.2. Composition analysis of immune cells

CIBERSORT is a robust algorithm for determining the proportions of immune cells in a single sample by using gene expression profiles [14] and this algorithm is used to assess the levels of immune cells in whole peripheral blood sample and parotid tissue from patients and controls. 1000 was set as the number of permutations to assure the accuracy of the results. *P* < 0.05 was set as the cutoff value.

### 2.3. Construction of the co-expression modules

The co-expression modules were constructed by using the expression of 7849 genes whose variance was ranked at the top quarter among all the 31,396 genes detected by microarray via the ‘WGCNA’ R package [17]. In our study, 0.8 was set as the correlation coefficient threshold, while 30 was chosen to be the minimum number of genes in the modules.

### 2.4. Functional enrichment analysis

To investigate the function of genes in the modules mostly related to B cells, we referred to Metascape database [23] (https://metascape.org/) to perform the Gene Ontology (GO) analysis and pathway analysis. *P* < 0.05 was set as the cutoff value.

### 2.5. Identification of differentially expressed genes (DEGs)

We identified DEGs between samples from patients and controls via GEO2R tool in the NCBI (https://www.ncbi.nlm.nih.gov/geo/geo2r/). The GEO2R is an online tool set in the NCBI website to help researchers identify DEGs among different samples and is widely used in bioinformatic analysis. *P* value <0.05 and fold change >1 were set as the cutoff criteria.

### 2.6. Identification of hub genes

We firstly identified the intersections between DEGs and genes in the modules related to B cells. Search tool for recurring instances of neighboring genes (String) database (https://string-db.org/) was used to construct interaction networks of these intersecting genes. The interactions between these genes can be obtained by simply inputting the symbols into the search window on the website and then inputted into Cytoscape software for further analysis. Next, the hub genes of these gene interaction networks were further identified and visualized by Cytohubba app in the Cytoscape software [24].

### 2.7. GSEA analysis

The KEGG pathways enriched in the peripheral blood of pSS patients were identified by the GSEA method [25] and nominal P < 0.05 was set as the cutoff value. The reference gene sets file was downloaded from the GSEA website (https://pypi.org/project/gseapy/).

### 2.8. Construction of a diagnostic model for pSS

Multiple Logistic regression analysis was applied to construct a model based on the expression of hub genes for pSS diagnosis. Moreover, to obtain the optimized model, Least Absolute Shrinkage and Selection Operator (LASSO) regression analysis was performed via ‘glmnet’ R packages.

### 2.9. Statistical analysis

All statistical analyses in the present study were performed with R version 4.0.1 and *P* value <0.05 was considered statistically significant.

## 3. Results

B cells have been identified to be significantly involved in the pathogenesis of pSS. The levels of B cell subpopulations in the whole peripheral blood and parotid tissue of pSS patients have been evaluated and it has been found that the best-characterized change of B cells in pSS patients is the decreased number of memory B cells in the peripheral blood, in addition, the elevated level of B cells in parotid tissue has also been identified [7–12]. However, to date, the mechanism underlying this change in B cell level is still unknown. Therefore, to investigate the underlying mechanism, we performed a series of bioinformatic analyses, including CIBERSORT, WGCNA and GSEA analysis to identify key genes and pathways involved in this phenomenon. Furthermore, we constructed a diagnostic model for pSS by using the expression pattern of hub genes identified via multiple Logistic regression. The exact results of our study are presented below.

### 3.1. Levels of B cells in samples from pSS patients and controls

**Figure 1.**
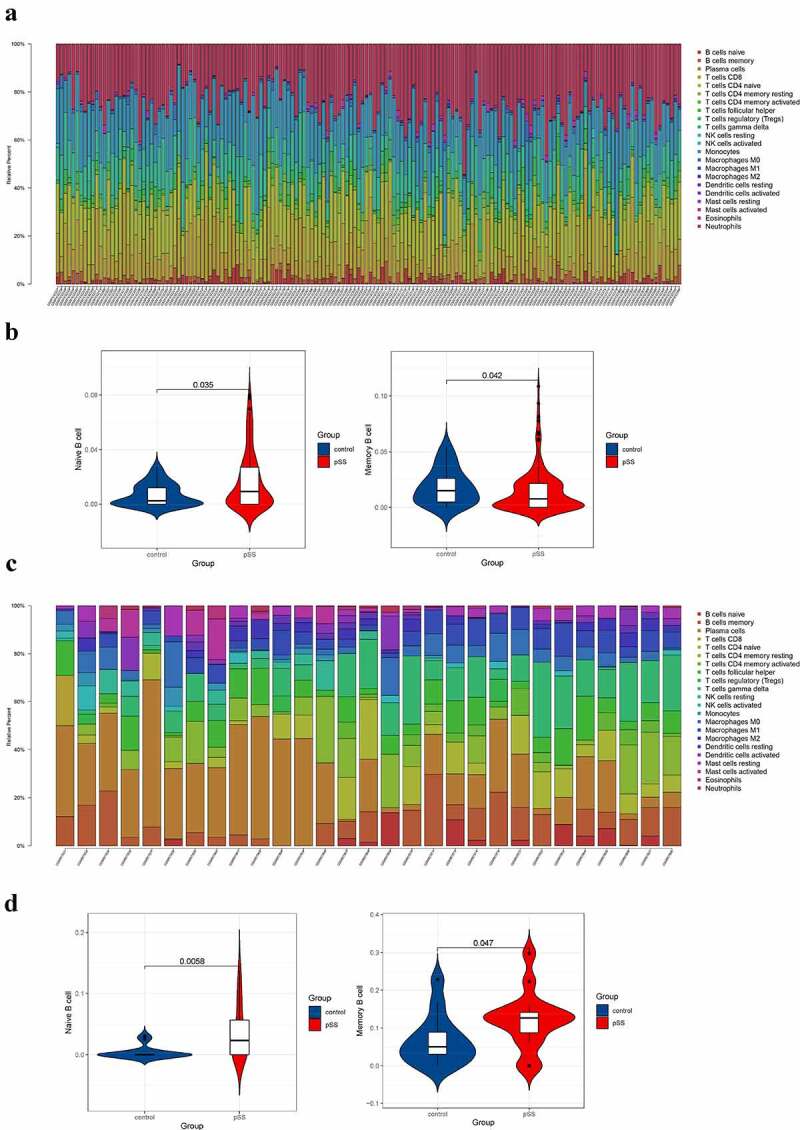
Composition analysis of immune cells of peripheral blood and parotid samples from pSS patients and controls. (a) The visualization of levels of immune cells in the peripheral blood from pSS patients and controls. (b) The violin plot showed an elevated level of naïve B cells and a decreased level of memory B cells in the peripheral blood of pSS patients (P < 0.05). (c) The visualization of levels of immune cells in the parotid tissues from pSS patients and controls. (d) The violin plot showed elevated levels of naïve B cells and memory B cells in the parotid tissues of pSS patients (P < 0.05)

The results showed that the level of naïve B cell in blood of pSS patients was higher than that of controls (*P* < 0.05) and that the level of memory B cell in blood of pSS patients was lower than that of controls (*P* < 0.05) (Figure 1(b)), which were consistent with previous studies [9–13]. For parotid tissue, the level of naïve B cell of pSS patients was significantly higher than that of controls and interestingly, the level of memory B cell in patients was higher than that in controls (Figure 1(d)). The results of CIBERSORT in GSE66795 and GSE40611 were set in Table S1.

### 3.2. Construction of co-expression modules in whole peripheral blood and parotid tissue

**Figure 2.**
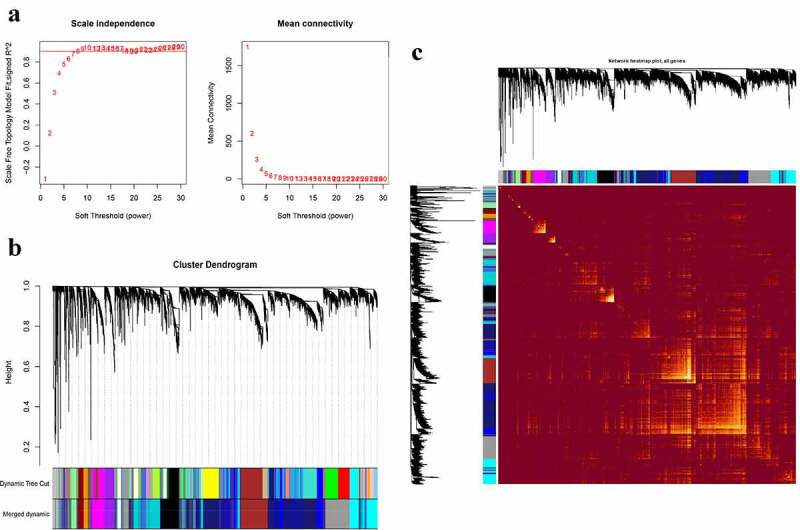
Construction of co-expression modules in the whole peripheral blood from pSS patients and controls. (a) Screening for power values. When 7 is chosen as the soft threshold, the scale independence is greater than 0.8 and the mean connectivity is lesser than 100. (b) Clustering dendrogram of co-expression modules. (c) The network heatmap plot of the modules

**Figure 3.**
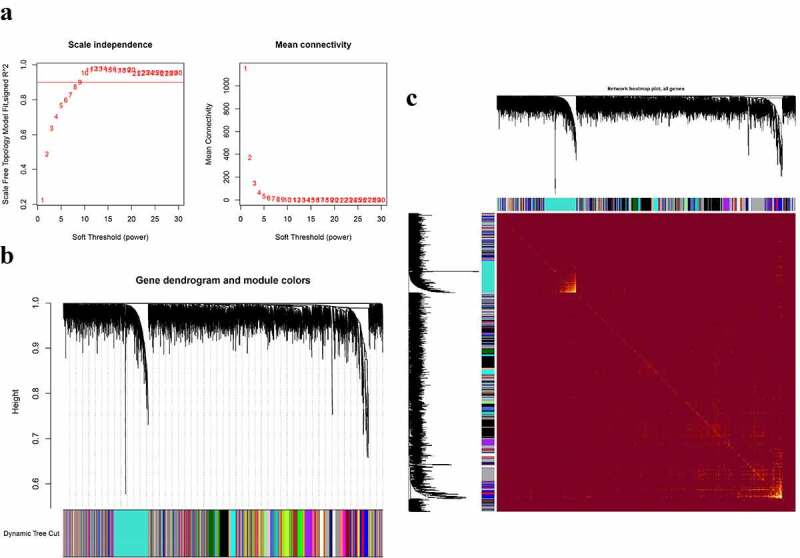
Construction of co-expression modules in the parotid tissues from pSS patients and controls. (a) Screening for power values. When 9 is chosen as the soft threshold, the scale independence is greater than 0.8 and the mean connectivity is lesser than 100. (b) Clustering dendrogram of co-expression modules. (c) The network heatmap plot of the modules

The co-expression modules were constructed via the WGCNA method (Figures 2 and 3). Figures 2(a,b) and 3(a,b) shows the quality control process. Network heatmap plot of the modules is shown in Figures 2(c) and 3(c). For the analysis of peripheral blood, we chose 7 as the soft-thresholding power (Figure 2(a)). For the analysis of parotid tissue, we chose 9 as the soft-thresholding power (Figure 3(a)).

### 3.3. Identification of co-expression modules significantly associated with B cells

**Figure 4.**
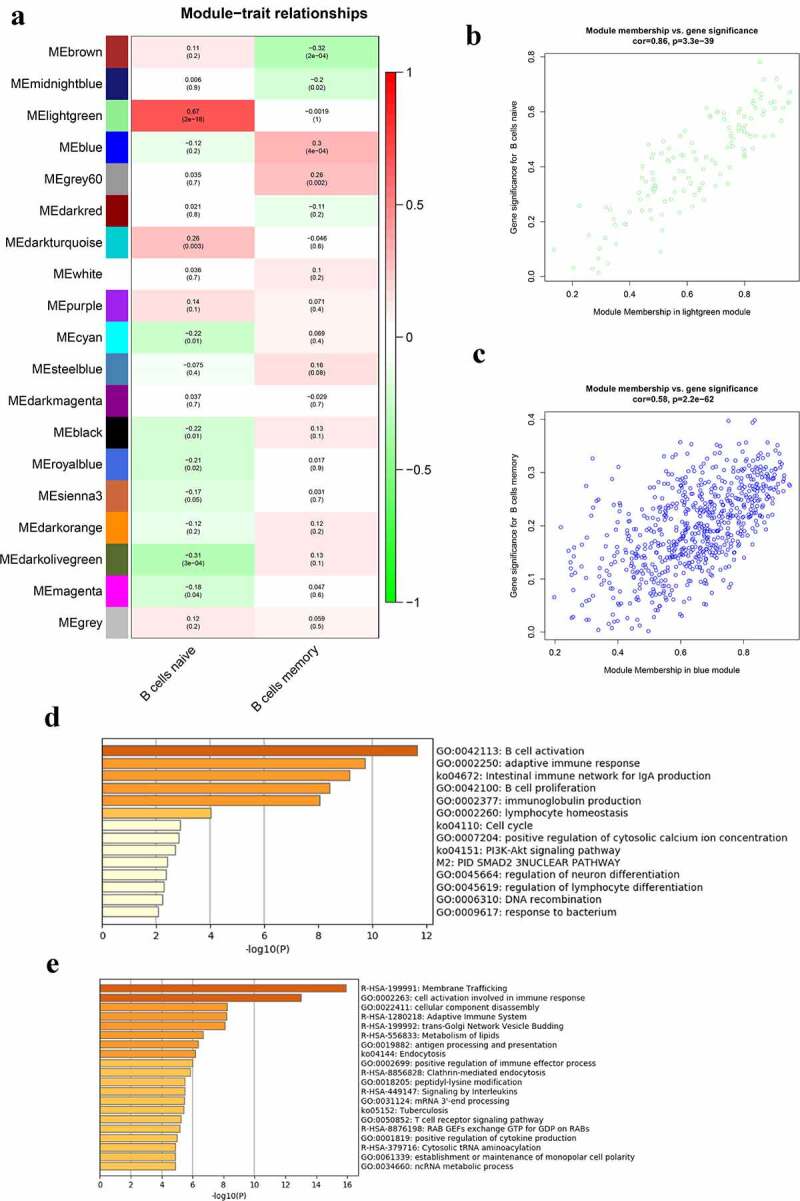
Co-expression modules mostly related to B cells in the whole peripheral blood in pSS. (a) Heatmap plot of the correlation between modules and B cells in the whole peripheral blood. (b) The gene significance for naïve B cells in the lightgreen module (One dot in the picture represents one gene in the lightgreen module.). (c) The gene significance for memory B cells in the blue module. (d) Functional enrichment in the lightgreen module. (e) Functional enrichment in the blue module

The correlation between modules and B cells in the whole peripheral blood is presented in Figure 4(a). The lightgreen module was mostly related to naïve B cells and the blue module was mostly related to memory B cells in the peripheral blood. There were 125 genes in the lightgreen module and 667 genes in the blue module. The genes in the lightgreen module and the blue module were set in Table S4. Figure 4(b,c) shows the significance of genes in the lightgreen and blue modules for naïve B cells and memory B cells, respectively. The GO terms and pathways enriched in the lightgreen and blue modules are shown in Figure 4(d,e), respectively. The top three GO terms significantly enriched in lightgreen module were B cell activation, adaptive immune response, and B cell proliferation. Moreover, GO terms and pathways enriched in the blue module included cell activation involved in immune response, cellular components disassembly, antigen processing and presentation, membrane trafficking, and adaptive immune system.

**Figure 5.**
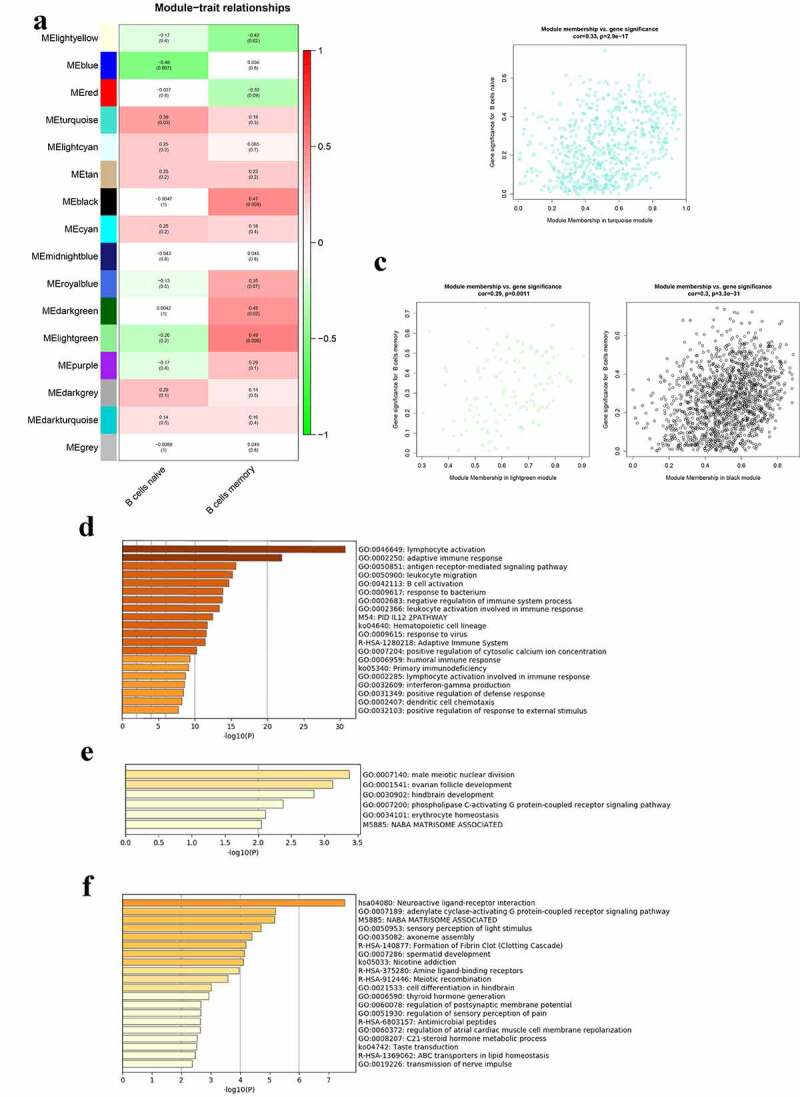
Co-expression modules mostly related to B cells in the parotid tissues in pSS. (a) Heatmap plot of the correlation between modules and B cells in the parotid tissue. (b) The gene significance for naïve B cells in the turquoise module. (c) The gene significance for memory B cells in the lightgreen (left) and black (right) modules. (d) Functional enrichment in the turquoise module. (e) Functional enrichment in the lightgreen module. (f) Functional enrichment in the black module

The correlation between modules and B cells in the parotid tissue is presented in Figure 5(a). The turquoise module was mostly related to naïve B cells, and the lightgreen and black modules were mostly related to memory B cells in the parotid tissues. There were 573 genes in the turquoise module, 115 genes in lightgreen module and 1381 genes in the black module. The genes in these three modules were stored in Table S5. Figure 5(b,c) shows the significance of genes in the turquoise, lightgreen, and black modules for naïve B cells and memory B cells, respectively. The GO terms and pathways enriched in turquoise, lightgreen, and black modules are shown in Figure 5(d–f), respectively. GO terms significantly enriched in the turquoise module were lymphocyte activation, adaptive immune response, antigen receptor mediated signaling pathway, leukocyte migration, and B cell activation.

### 3.4. Hub genes involved in the levels of B cells in pSS

**Figure 6.**
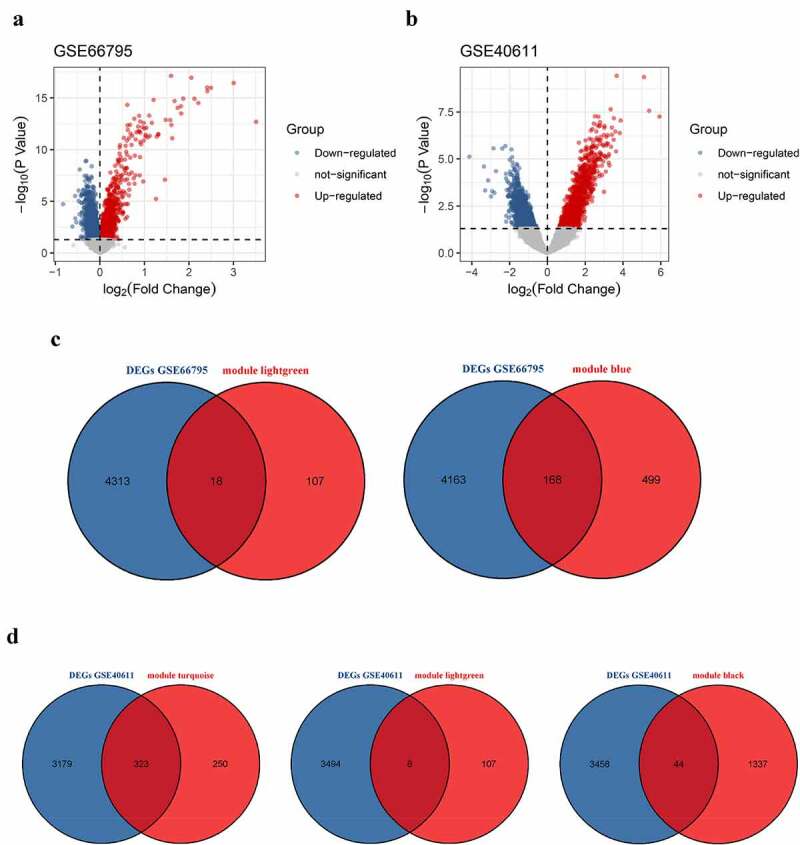
Identification of differentially expressed genes (DEGs). (a) DEGs in GSE66795. (b) DEGs in GSE40611. (c) The numbers of intersecting genes between DEGs in GSE66795 and genes in the lightgreen module (left) and blue module (right). (d) The numbers of intersecting genes between DEGs in GSE40611 and turquoise, lightgreen and black module

**Figure 7.**
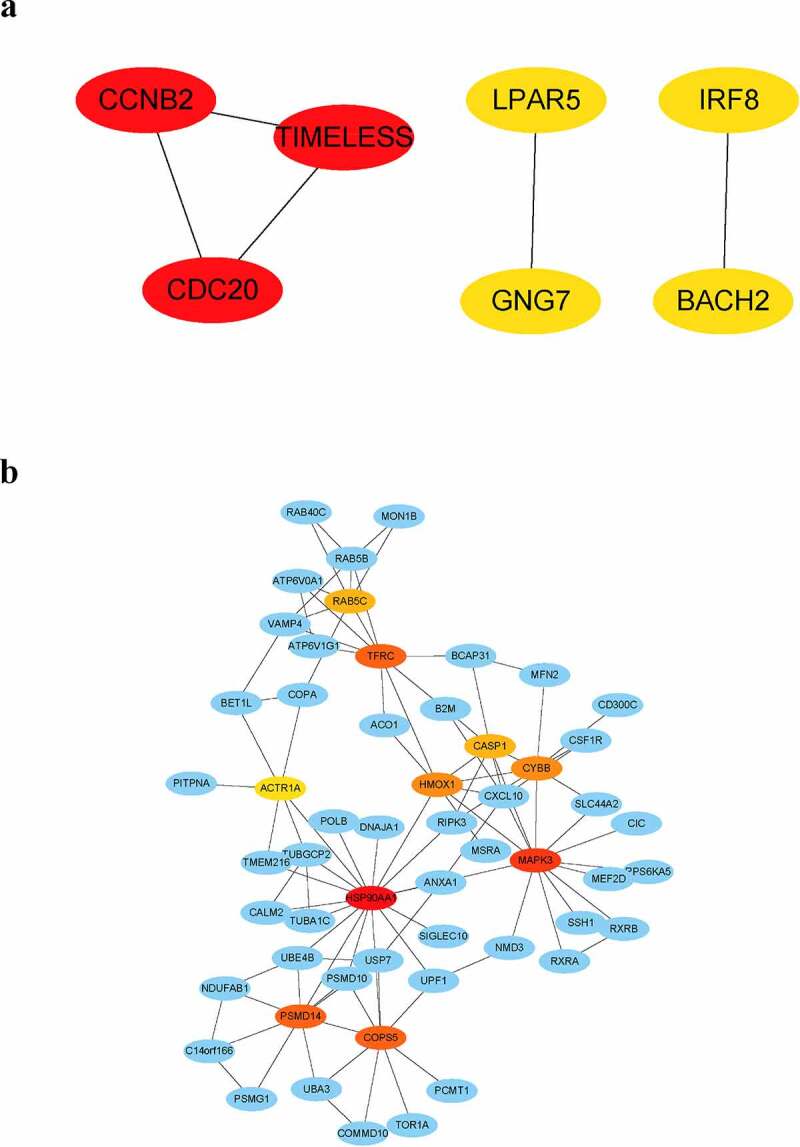
Visualization of hub genes involved in the levels of B cells in the whole peripheral blood of pSS patients. (a) The top 7 hub genes involved in the increased level of circulating naïve B cells. (b) The top 10 hub genes involved in the decreased level of circulating memory B cells

**Figure 8.**
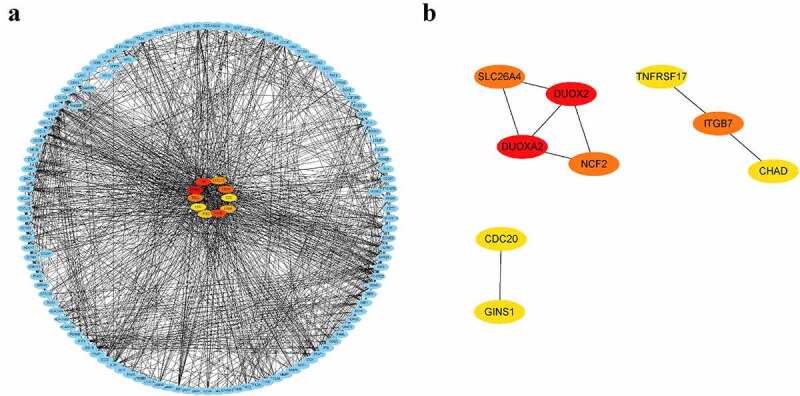
Visualization of hub genes associated with the levels of B cells in the parotid tissue of pSS patients. (a) The top 10 hub genes associated with the increased level of naïve B cells in parotid tissue. (b) The top 9 hub genes related to the elevated level of memory B cells in parotid tissue

DEGs in peripheral blood and parotid tissue between pSS patients and controls are shown in Figure 6(a,b), respectively. These DEGs were also set up in Table S3. The number of intersecting genes between DEGs in peripheral blood and genes in the lightgreen module and blue module is presented in Figure 6(c). Moreover, Figure 6(d) shows the number of intersecting genes between DEGs in parotid tissue and turquoise, lightgreen, and black modules. Figure 7 shows the hub genes involved in the levels of circulating B cells of pSS patients, and the visualization of hub genes associated with the levels of B cells in parotid tissue is set up in Figure 8. Hub genes involved in the increased level of naïve B cells in peripheral blood of patients included CCNB2, TIMELESS, CDC20, GNG7, LPAR5, BACH2 and IRF8. Hub genes involved in the decreased level of memory B cells in peripheral blood of patients included HSP90AA1, MAPK3, TFRC, PSMD14, COPS5, HMOX1, CYBB, RAB5C, CASP1 and ACTR1A. Hub genes associated with the elevated level of naïve B cells in parotid tissue of patients with pSS included PTPRC, TNF, CCR5, CD19, SELL, CXCL10, CD69, IFNG, LCK, and CD2. Furthermore, hub genes related to the elevated level of memory B cells in parotid tissue of patients with pSS included DUOXA2, DUOX2, NCF2, ITGB7, SLC26A4, TNFRSF17, GINS1, CHAD, and CDC20.

### 3.5. Validation of expression of hub genes in GSE51092 dataset

**Figure 9.**
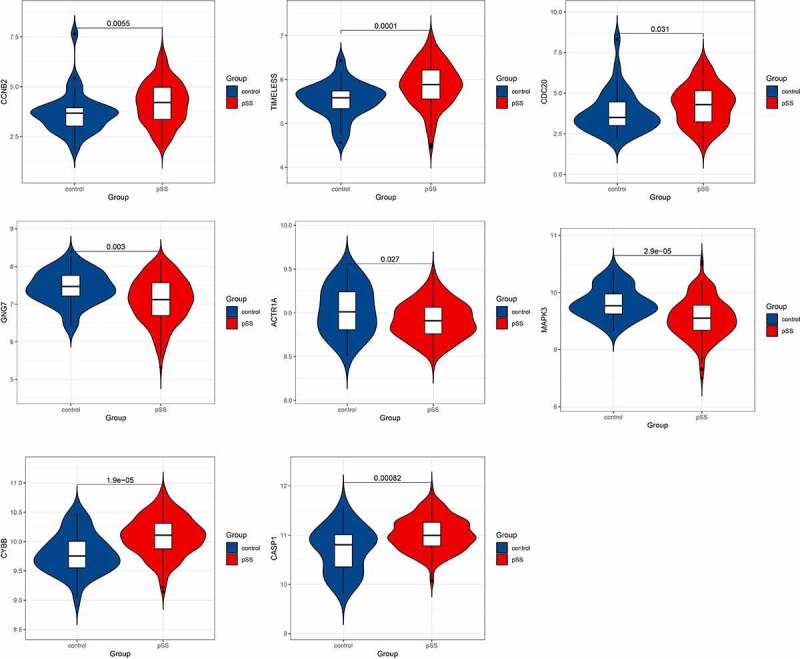
Hub genes showing significantly differential expression in GSE51092 dataset

After identifying the hub genes involved in the levels of naïve B cells and memory B cells in the whole peripheral blood of pSS patients, we validated the expression of these hub genes in another dataset GSE51092. The hub genes that showed significant differential expression in GSE51092 included CCNB2, TIMELESS, CDC20, GNG7, ACTR1A, MAPK3, CYBB and CASP1 (Figure 9).

### 3.6. GSEA of pSS

**Figure 10.**
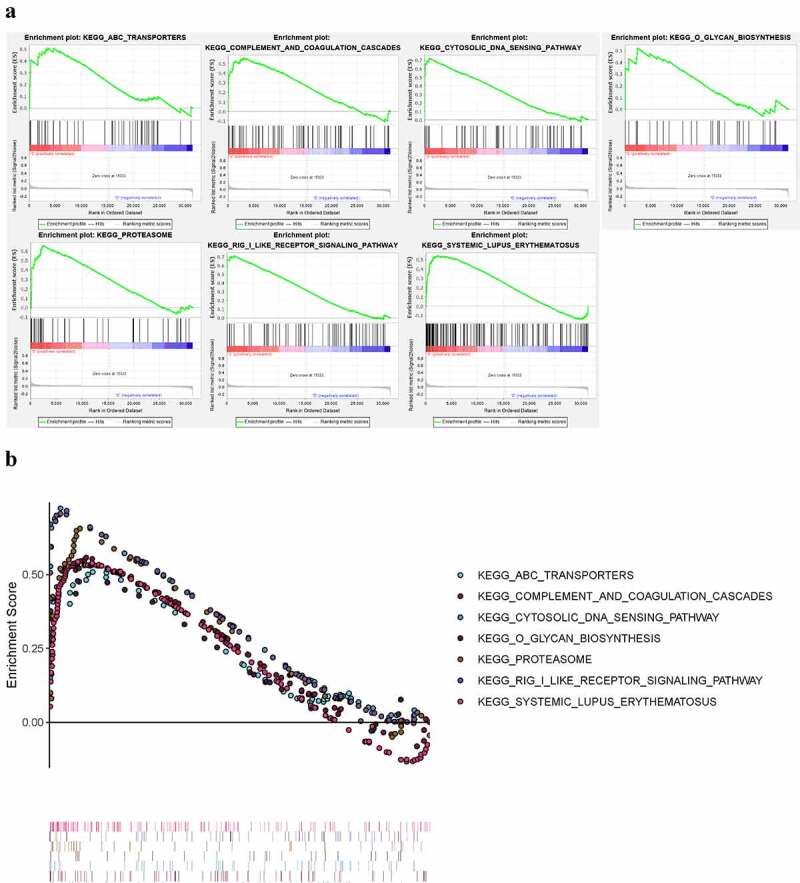
KEGG pathways significantly enriched in peripheral blood of pSS patients via GSEA (nominal *P* < 0.05)

We applied GSEA to identify KEGG pathways significantly enriched in the peripheral blood of pSS (nominal *P* < 0.05) (Figure 10). The results showed that ABC-TRANSPORTERS, COMPLEMENT-AND-COAGULATION-CASCADES, CYTOSOLIC-DNA-SENSING-PATHWAY, O-GLYCAN-BIOSYNTHESIS, PROTEASOME, RIG-I-LIKE-RECEPTOR-SIGNALING-PATHWAY, SYSTEMIC-LUPUS-ERYTHEMATOSUS pathways were enriched in pSS.

**Figure 11.**
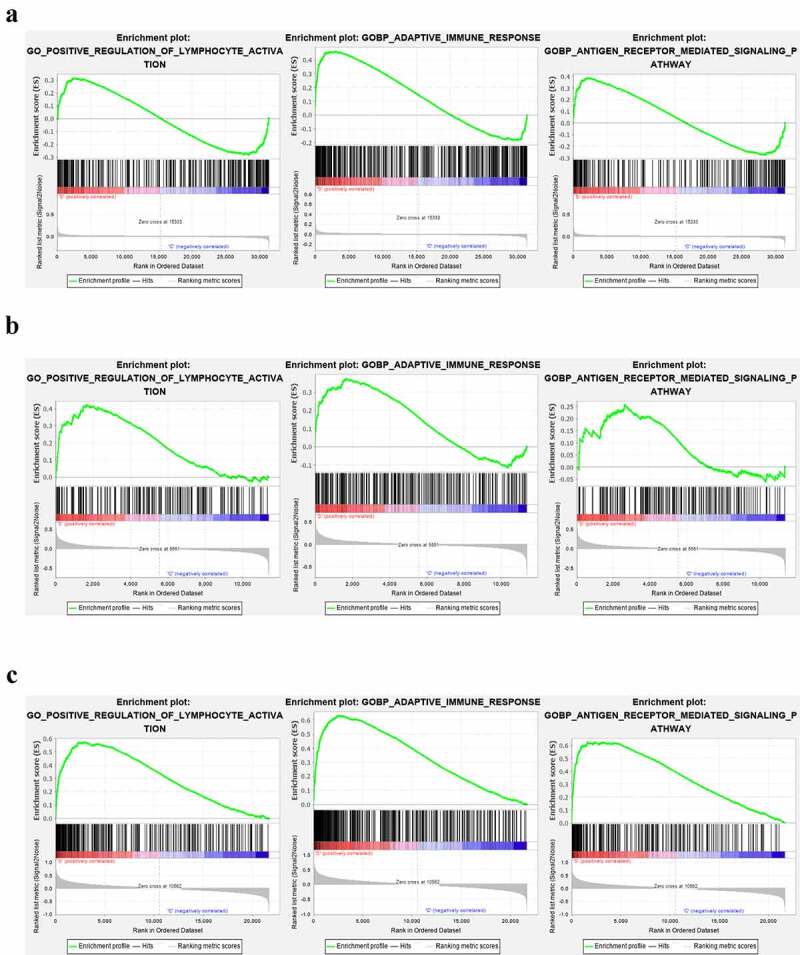
The GSEA analysis of lymphocyte activation, adaptive immune response and antigen receptor pathway in GSE66795, GSE51092 and GSE40611. (a) The adaptive immune response pathway was found to be significantly enriched in the peripheral blood of pSS patients in the GSE66795 dataset. (b) The adaptive immune response and the positive regulation of lymphocyte activation pathways were significantly enriched in the peripheral blood of pSS patients in GSE51092. (c) All of these three pathways were significantly enriched in the parotid tissue of pSS patients in GSE40611

We further performed GSEA analysis of lymphocyte activation, adaptive immune response, and antigen receptor pathway in the GEO datasets (GSE66795, GSE40611, and GSE51092). In the GSE66795 dataset, only the adaptive immune response pathway was found to be significantly enriched in the peripheral blood of pSS patients (nominal p = 0.032 < 0.05) (Figure 11(a)). In the GSE51092 dataset, both adaptive immune response and the positive regulation of lymphocyte activation pathways were significantly enriched in the peripheral blood of pSS patients with nominal p = 0.035, 0.0018, respectively (Figure 11(b)). As for the GSE40611, the results showed that all these three pathways were significantly enriched in the parotid tissue of pSS patients with nominal p = 0 (Figure 11(c)). These results indicated the involvement of the activation of lymphocytes and adaptive immune response in the development of pSS.

### 3.7. Construction and validation of the gene-based diagnostic model for pSS

**Figure 12.**
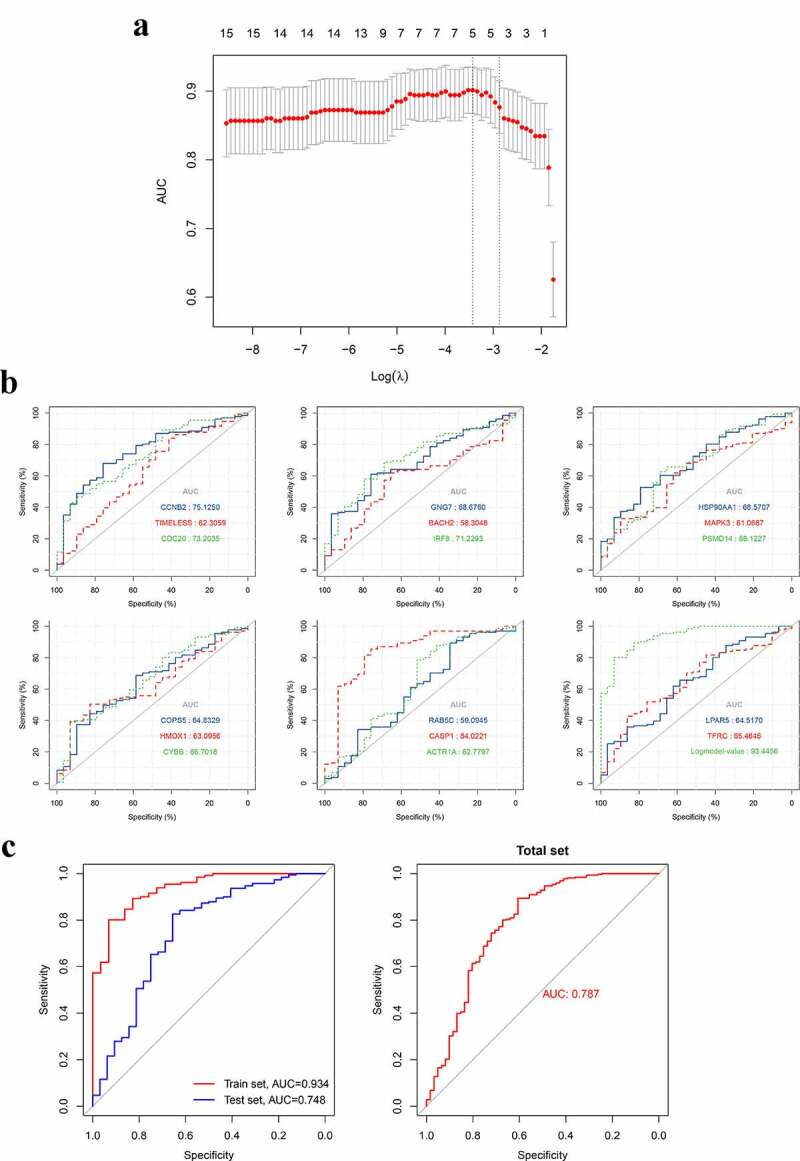
The construction and assessment of the gene-based diagnostic model. A. The screening process of the models (via LASSO regression analysis). B. ROC curve and AUC value of hub genes in the training set. C. The ROC curve and AUC value of the diagnostic model in train set GSE66795, the test set GSE51092 and the total set. LASSO, Least absolute shrinkage and selection operator

After identifying the hub genes in the blood that are significantly associated with blood naïve B and memory B cells, we then assessed the diagnostic values of these genes via ROC curve and area under curve (AUC) (Figure 12(b)). Furthermore, we tried to construct a diagnostic model for pSS by using the expression patterns of these hub genes, and ultimately, an optimized diagnostic model consisting of five genes was established (Figure 12(a), Table 1). Moreover, we assessed the diagnostic value of this model in a test set GSE51092, and the AUC indicated that this model is a robust tool for assisting the diagnosis of pSS (Figure 12(c), Table S2). The probability for having pSS was calculated as follows:

$$Probability=\frac{e^{\left(\beta 0+\beta X1+\beta X2+\ldots +\beta Xn\right)}}{1+e^{\left(\beta 0+\beta X1+\beta X2+\ldots +\beta Xn\right)}}$$

**Table 1.** The genes in the diagnostic model identified by Logistic regression analysis

Gene symbol	Gene name	Coef	OR value
(Intercept)	-	−30.2744	-
TIMELESS	Timeless Circadian Regulator	1.349366	3.85743
CDC20	Cell Division Cycle 20	1.040184	2.82922
IRF8	Interferon Regulatory Factor 8	−2.03766	0.13003
HMOX1	Heme Oxygenase 1	0.433039	1.54188
CASP1	Caspase 1	3.772143	43.38006

*Intercept = β0, the constant in the formula. ‘Coef’ represents the coefficients of genes.

where X represents the expression of a gene included in this diagnostic model. β is the coefficient of a gene in the model. β0 is a constant. As for the OR value of genes, according to the multiple Logistic regression, the OR value of a gene can be calculated as the following formula:

$$ORi=e^{\beta }$$

where i represents a gene in the model and β is the coefficient of this gene in the model. The threshold of probability that we set of this model was 0.5, which means that, when the value of one person calculated by the formula was >0.5, this person would be considered to be a pSS patient.

## 4. Discussion

B cell populations are generally considered to be remarkably involved in the development and progression of pSS. To date, we evaluated the levels of B cells in the whole peripheral blood and parotid tissue (a target organ of pSS) from patients with pSS by CIBERSORT and identified hub genes involved in the altered levels of B cells in pSS patients by WGCNA for the first time. We also identified DEGs between blood samples and parotid tissue from pSS patients and healthy controls. Through these analyses, we established gene co-expression modules and identified hub genes in peripheral blood and parotid tissue that were associated with levels of B cells in pSS, which might provide some new insights into the development and progression of pSS.

Biological signs of B cell activation have been identified in pSS patients. However, the underlying mechanism is not clear for now. In our study, for peripheral blood, we obtained co-expression modules via WGCNA and found that the lightgreen module was most associated with the increased level of naïve B cells of pSS patients. Genes in lightgreen module were significantly enriched in B cell activation and B cell proliferation GO terms, indicating that these genes may play an important role in the development of pSS. We further identified 7 hub genes involved in the increased level of circulating naïve B cells of pSS patients. Among them, CCNB2, TIMELESS, CDC20 and GNG7 showed differential expression levels between patients with pSS and healthy controls in GSE66795 and the expression patterns of them were validated in another dataset GSE51092. The roles of these four genes in pSS have not been discussed. CCNB2, cyclin B2, encodes a protein that belongs to cyclin family and is essential to the cell cycle regulation. Therefore, the high expression of CCNB2 in peripheral blood may be an indicator of fast proliferation of naïve B cells in pSS. As for TIMELESS, Timeless Circadian Regulator, it takes a part in cell survival after damage or stress and also plays a role in the circadian rhythm autoregulatory loop. Changes in this gene or its expression may promote multiple cancers such as prostate cancer and lung cancer [26,27] and mental disorders [28]. The overexpression of TIMELESS in peripheral blood of pSS patients was found in GSE66795 and validated in an external dataset GSE51092. Notably, pSS patients have been found to suffer from sleep disturbances [29]. Therefore, TIMELESS might not only be associated with naïve B cells but also related to the sleep deficit and mental symptoms of pSS patients. CDC20 can interact with CCNB2 to regulate cell cycle; therefore, it may regulate the proliferation of naïve B cells. GNG7, G protein subunit gamma 7, is a protein encoding gene and the reduced expression of it is associated with squamous cell carcinoma [30] and esophageal cancer [31]. The low expression level of GNG7 is also found in the whole peripheral blood of pSS patients in our study, but the mechanism of this gene in pSS is still unknown and needed to be further investigated.

For parotid tissue, we found that the turquoise module was significantly associated with the increased level of naïve B cells of pSS patients. Genes in the turquoise module were significantly enriched in lymphocyte activation, adaptive immune response, antigen receptor mediated signaling pathway, leukocyte migration, and B cell activation GO terms, which indicated that these genes may be associated with the activation of naïve B cells and the migration of circulating naïve B cells into the parotid tissue in pSS.

Previous studies have found the decreased number of circulating memory B cells of pSS patients and the increased number of memory B cells in the salivary glands of pSS patients [12,13]. The results of CIBERSORT were consistent with these findings. However, the mechanism underlying this phenomenon is still not clear. In our study, the blue module was found to be most associated with the decreased level of circulating memory B cells of pSS patients and the black module was most related to the increased level of memory B cells in parotid tissue of pSS patients. Therefore, genes in these two modules may be associated with the altered level of memory B cells in pSS patients mentioned above. We further identified 10 hub genes involved in the decreased level of circulating memory B cells of pSS patients. Among them, ACTR1A, MAPK3, CYBB and CASP1 showed differential expression levels between patients with pSS and controls in GSE66795 and the expression patterns of them were validated in another dataset GSE51092. However, the roles of these four genes in pSS have not been discussed. ACTR1A (actin-related protein 1A) encodes a subunit of dynactin, which is involved in multiple cellular functions, such as endoplasmic reticulum (ER)-to-Golgi transport and spindle formation. Furthermore, ACTR1A has been identified as a novel regulator of Toll-like receptor 2 (TLR2)-mediated immune signaling pathways [32]. TLR2 is a pattern recognition receptor that can initiate pro-inflammatory responses by the cell through interacting with other proteins. Therefore, ACTR1A may take part in the development of pSS through TLR2-mediated signaling pathways. MAPK3 (mitogen-activated protein kinase 3) is a member of the MAP kinase family. The lower expression of this gene in pSS patients was found and validated in our study, which may indicate the involvement of MAPK signaling cascade in the development of pSS. CYBB (cytochrome b-245 beta chain) is a component of cytochrome b-245, which has been found to be associated with immunodeficiency. The overexpression of CYBB was identified and validated in our study, which may indicate the overactivity of immune response in pSS patients. The protein encoded by CASP1 (caspase 1) belongs to the caspase family and notably, the sequential activation of caspases plays a central role in cell apoptosis. Moreover, CASP1 has been identified to have the ability to activate interleukin-1 [33], which is a cytokine associated with multiple processes, such as inflammation and septic shock. The observed overexpression of CASP1 in our study may suggest the involvement of cell apoptosis-related pathways in the decreased level of memory B cells in the peripheral blood of pSS patients. However, the exact mechanisms underlying the influence of these four genes over the level of circulating memory B cells of pSS patients are still unclear and need to be further investigated.

Hub genes involved in the increased level of memory B cells in parotid tissue of pSS patients were also identified, including DUOXA2, DUOX2, NCF2, ITGB7, SLC26A4, TNFRSF17, GINS1, CHAD and CDC20. DUOXA2, DUOX2, NCF2 and SLC26A4 can form a network through the interactions between them. DUOXA2 (dual-oxidase maturation factor 2) is necessary for maturation of functional dual oxidase 2 (DUOX2) and previous studies have suggested that the DUOX2/DUOXA2 pathways may be associated with the intestinal inflammation [34,35] and the immune responses in airway epithelia [36]. Therefore, the interactive network of DUOXA2, DUOX2, NCF2 and SLC26A4 may be involved in the local immune response in parotid tissue of pSS patients. For GINS1 (GINS complex subunit 1), the GINS complex is known to be essential for the initiation of eukaryotic DNA replication and diseases associated with it included immunodeficiency and neutropenia [37]. Furthermore, it can interact with CDC20 and may influence the cell cycle of memory B cells in parotid tissue of pSS patients together with CDC20. TNFRSF17 (TNF receptor superfamily member 17) belongs to the TNF-receptor superfamily and is found to be preferentially expressed in mature B lymphocytes, and may play an important role in B cell maturation and autoimmune response [38–40]. ITGB7 (integrin subunit beta 7) is a member of the integrin superfamily. ITGB7 plays a role in leukocyte adhesion and the migration of lymphocytes and takes part in the formation of a homing receptor for migration of B cells to the intestinal mucosa and Peyer’s patches [41]. Therefore, it may involve in the increased level of memory B cells in parotid tissue of pSS patients by stimulating the migration of memory B cells. As for CHAD (chondroadherin), it is a cartilage matrix protein, which is considered to be involved in mediating adhesion of isolated chondrocytes [42].

The diagnostic value of hub genes in the blood was assessed and then a robust diagnostic model for pSS was established and validated on an external dataset via using the expression patterns of five hub genes (TIMELESS, CDC20, IRF8, HMOX1 and CASP1). This diagnostic model may assist doctors in diagnosing pSS through the expression analysis of these 5 genes in the blood.

Although our study is the first to investigate key genes and pathways influencing the levels of B cells in pSS using CIBERSORT and WGCNA, still, our study has some limitations. First, the exact mechanisms underlying the effects of the hub genes involved were not studied in our study and needed to be further investigated. Secondly, the results in this study are obtained only by bioinformatic analysis and need to be further validated by wet experiments. Moreover, the target organ of pSS discussed in this study was only the parotid tissue. Thus, further investigation is needed in other target organs, such as lachrymal glands.

## 5. Conclusion

In conclusion, through a combination of multiple bioinformatic methods, our study identified hub genes and key pathways significantly associated with the activation of B cells and the altered levels of B cells in pSS. And we established a diagnostic model for pSS based on the expression of five genes in the peripheral blood, which may assist clinicians in diagnosing pSS. However, the diagnostic value of this model still needs to be tested in a large-scale investigation. The results of our study may provide some new insights into the behaviors of B cells in pSS and the pathogenesis of pSS.

## Supplementary Material

Supplemental Material
